# Expression patterns of signaling lymphocytic activation molecule family members in peripheral blood mononuclear cell subsets in patients with systemic lupus erythematosus

**DOI:** 10.1371/journal.pone.0186073

**Published:** 2017-10-11

**Authors:** Maria P. Karampetsou, Denis Comte, Katalin Kis-Toth, Vasileios C. Kyttaris, George C. Tsokos

**Affiliations:** 1 Division of Rheumatology, Beth Israel Deaconess Medical Center, Harvard Medical School, Boston, Massachusetts, United States of America; 2 Division of Immunology and Allergy, Centre Hospitalier Universitaire Vaudois, Lausanne, Switzerland; IMAGINE, FRANCE

## Abstract

Genome-wide linkage analysis studies (GWAS) studies in systemic lupus erythematosus (SLE) identified the 1q23 region on human chromosome 1, containing the Signaling Lymphocytic Activation Molecule Family (SLAMF) cluster of genes, as a lupus susceptibility locus. The SLAMF molecules (SLAMF1-7) are immunoregulatory receptors expressed predominantly on hematopoietic cells. Activation of cells of the adaptive immune system is aberrant in SLE and dysregulated expression of certain SLAMF molecules has been reported. We examined the expression of SLAMF1-7 on peripheral blood T cells, B cells, monocytes, and their respective differentiated subsets, in patients with SLE and healthy controls in a systematic manner. SLAMF1 levels were increased on both T cell and B cells and their differentiated subpopulations in patients with SLE. SLAMF2 was increased on SLE CD4+ and CD8+ T cells. The frequency of SLAMF4+ and SLAMF7+ central memory and effector memory CD8+ T cells was reduced in SLE patients. Naïve CD4+ and CD8+ SLE T cells showed a slight increase in SLAMF3 levels. No differences were seen in the expression of SLAMF5 and SLAMF6 among SLE patients and healthy controls. Overall, the expression of various SLAMF receptors is dysregulated in SLE and may contribute to the immunopathogenesis of the disease.

## Introduction

Systemic Lupus Erythematosus (SLE) is a multisystem autoimmune disease characterized by a loss of tolerance to self antigens, the production of autoantibodies and inflammation of multiple organs [1–3]. Genome wide association studies (GWAS) in human SLE have identified the 1q23 region on chromosome 1, which contains the *SLAMF* (Signaling Lymphocytic Activation Molecule Family) cluster of genes, as a susceptibility locus for lupus [4, 5]. In addition, the syntenic genomic region 1H3 has been described to be associated with autoimmune manifestations in three different murine models of spontaneous lupus, namely the NZB/W F1, NZM and BXSB strains [6–8]. Specific SLAMF variants and single nucleotide polymorphisms have been associated with autoimmune diseases, such as rheumatoid arthritis, and/or specific lupus manifestations, such as neuropsychiatric and renal disease, thus further underscoring the potential involvement of the SLAMF molecules in autoimmunity [9–12].

The *SLAMF* gene cluster encodes seven co-regulatory receptors: SLAMF1 (CD150, SLAM), SLAMF2 (CD48), SLAMF3 (CD229, Ly9), SLAMF4 (CD244, 2B4), SLAMF5 (CD84), SLAMF6 (CD352, NTBA, Ly108) and SLAMF7 (CD319, CRACC, CS1). SLAMF8 (CD353, BLAME) and SLAMF9 (CD84-H1) are located outside, but in close proximity to the *SLAMF* locus. SLAMF molecules are mainly expressed on hematopoietic cells. They are type I transmembrane glycoprotein cell surface receptors and they belong to the CD2 superfamily of immunoglobulin domain-containing molecules. The extracellular region of the SLAMF members is characterized by the presence of one variable (V)-Ig like and by one constant (C2)-Ig like domain, with the exception of SLAMF3 which is composed of four Ig-like domains (two variable and two constant) [13, 14]. A unique feature of the SLAMF members is that they act as self-ligands and they interact in a homophilic manner, with the exception of SLAMF2 that associates with SLAMF4.

All SLAMF members, apart from SLAMF2, which is structurally a glycophosphatidylinositol (GPI) membrane anchor without cytoplasmic tail, and SLAMF9 [14], contain a cytoplasmic domain characterized by the presence of one to four intracellular switch motif amino acid sequences (ITSM). Upon SLAMF engagement, the ITSM sequence recruits with high affinity the SLAM-associated protein (SAP) or EAT-2 and mediates downstream signaling.

Previous limited reports suggested an altered expression of isolated SLAMF protein on the surface of T and/or B cells from SLE patients compared to healthy controls (SLAMF1[15], SLAMF3 [16], SLAMF4 [17, 18], SLAMF6 [19, 20] and SLAMF7 [18]). However, a more detailed characterization of all SLAMF in the same patient material and in immune cell subsets has not been reported.

In this communication we systematically assess the cell-surface expression of SLAMF1-7 on peripheral blood mononuclear cells isolated from patients with SLE and healthy controls subjects. More specifically we examined the expression of SLAMF molecules on T cells, B cells and monocytes and then further assessed SLAMF expression in a more detailed manner on the surface of differentiated CD4+T cells, CD8+ T cells and B cells subsets, in an attempt to identify new subpopulations that may contribute to the pathogenesis of SLE. We report aberrant expression of all but SLAMF5 and SLAMF6 in immune cell subsets from patients with SLE.

## Material and methods

### Human SLE and control cells

All patients with SLE (n = 48) included in this study were diagnosed according to the American College of Rheumatology classification criteria [21]. Patients with SLE were recruited from the Division of Rheumatology at Beth Israel Medical Center and provided written consent, as approved by the Institutional Review Board of Beth Israel Deaconess Medical Center, in compliance with Helsinki Declaration. Age-, sex-, and ethnicity-matched healthy individuals were chosen as controls (n = 12–20). Disease activity score for the patients with SLE was measured using the SLEDAI scoring system (Table 1)

**Table 1:** Characteristics of patients with SLE included in the study.

Characteristics of SLE patients	(N = 48)
Age—yr	
Median	42.5
Range	22–72
Gender	
Female—(%)	42 (87.5)
Male—(%)	6 (12.5)
Ethnicity	
Afro-american—(%)	14 (29.2)
Asian—(%)	3 (6.3)
Hispanic—(%)	6 (12.5)
Caucasian—(%)	25 (52.1)
SLE disease activity index (SLEDAI)	
Median	3.3
Range	(0–21)
Treatments	
Prednisone—(%)	31 (64.6)
Hydroxychloroquine—(%)	37 (77.1)
Mycophenolate Mofetil—(%)	19 (39.6)
Azathioprine—(%)	9 (18.8)
Methotrextate—(%)	2 (4.2)
I.V. Immunoglobulins—(%)	2 (4.2)
Belimumab—(%)	2 (4.2)
Tocilizumab—(%)	1 (2.1)
Abatacept—(%)	1 (2.1)

### Cell isolation

Peripheral blood was collected in heparin-lithium tubes from the study subjects. Peripheral blood mononuclear cells (PBMC) were enriched by density gradient centrifugation (Lymphocyte Separation Medium, Corning Life Sciences). PBMC were cryopreserved in liquid nitrogen until analysis.

### Antibodies

The following antibodies were used for FACS staining: PE/Cy7 conjugated anti-CD3 (clone UCHT1), PerCP conjugated anti-CD8 (clone RPA-T8), APC conjugated anti-CD45RA (clone HI100), Alexa Fluor 488 conjugated anti-CCR7 (clone G043H7), PE/Dazzle 594 conjugated anti-CD19 (clone HIB19), Alexa Fluor 700 conjugated anti-CD14 (clone HCD14), PerCP conjugated anti-CD19 (clone HIB19), Alexa Fluor 488 conjugated anti-CD20 (clone 2H7), Brilliant Violet 510 conjugated anti-CD38 (clone HB-7), PE/Cy7 conjugated anti-CD24 (clone ML5), Brilliant Violet 421 conjugated CD27 (clone M-T271), Alexa Fluor 647 conjugated anti-IgD (clone IA6-2), PE conjugated anti-SLAMF1/CD150 (clone A12), PE conjugated anti-SLAMF2/CD48 (clone BJ40), PE conjugated anti-SLAMF3/CD229 (clone HLy-9.1.25), PE conjugated anti-SLAMF4/CD244 (clone C1.7), PE conjugated anti-SLAMF5/CD84 (clone CD84.1.21), PE conjugated anti-SLAMF6/CD352 (clone NT-7), PE conjugated anti-SLAMF7/CD319 (clone 162), APC/Brilliant Violet 421/PE conjugated Mouse IgG1 Isotype Control (clone MOPC-21) were purchased from Biolegend. PerCP eFLuor 710 conjugated anti-CD4 (clone SK3) was purchased from eBioscience.

### Cell staining

Cryopreserved blood peripheral mononuclear cells were stained for dead cells using the Zombie Aqua or UV Fixable Viability Kit (Biolegend), and then labeled with the mentioned surface antibodies. Data were acquired on a LSR II SORP (5 lasers, BD Bioscience) and analyzed using FlowJo (version 10.0.8, FlowJo Enterprise).

### Statistics

Kruskal Wallis was performed followed by correction for multiple comparisons using the Holm-Sidak method. Statistical analyses were performed using GraphPad Prism (version 6). Statistical significance was reported as follows: *p<0.05, ** p<0.01, *** p<0.001.

## Results

We examined the expression of SLAMF members (SLAMF1-7) on the surface of PBMC isolated from SLE patients and age-, sex-, ethnicity- matched healthy controls. The SLAMF expression was initially assessed on total T cells, CD4+T cells, CD8+T cells, double negative T cells (DNT, CD3+CD4-CD8-), B cells and monocytes. By assessing the expression of CCR7 and CD45RA on T cells we were able to distinguish the following differentiated CD4+ and CD8+ T cells subsets: naïve (CCR7+CD45RA+), central memory (CM, CCR7+CD45RA-) and effector memory (EM, CCR7-CD45RA-), as well as terminally differentiated effector memory CD8+ T cells (TDEM, CD8+CCR7-CD45RA+) (S1A Fig). The various B cell subsets were identified based on the expression of CD27, IgD, CD24 and CD38: naïve B cells (CD27-IgD+), unswitched memory (USM, CD27+IgD+), switched memory (SM, CD27+IgD-) and double negative B cells (DNB, CD27-IgD-). By gating on naïve B cells and staining with CD24 and CD38 we were able to define transitional B cells (CD24^hi^CD38^hi^) and by gating on switched CD27+IgD- cells we defined CD24-CD38+ plasmablasts (S1B Fig).

### SLAMF1

**Fig 1:**
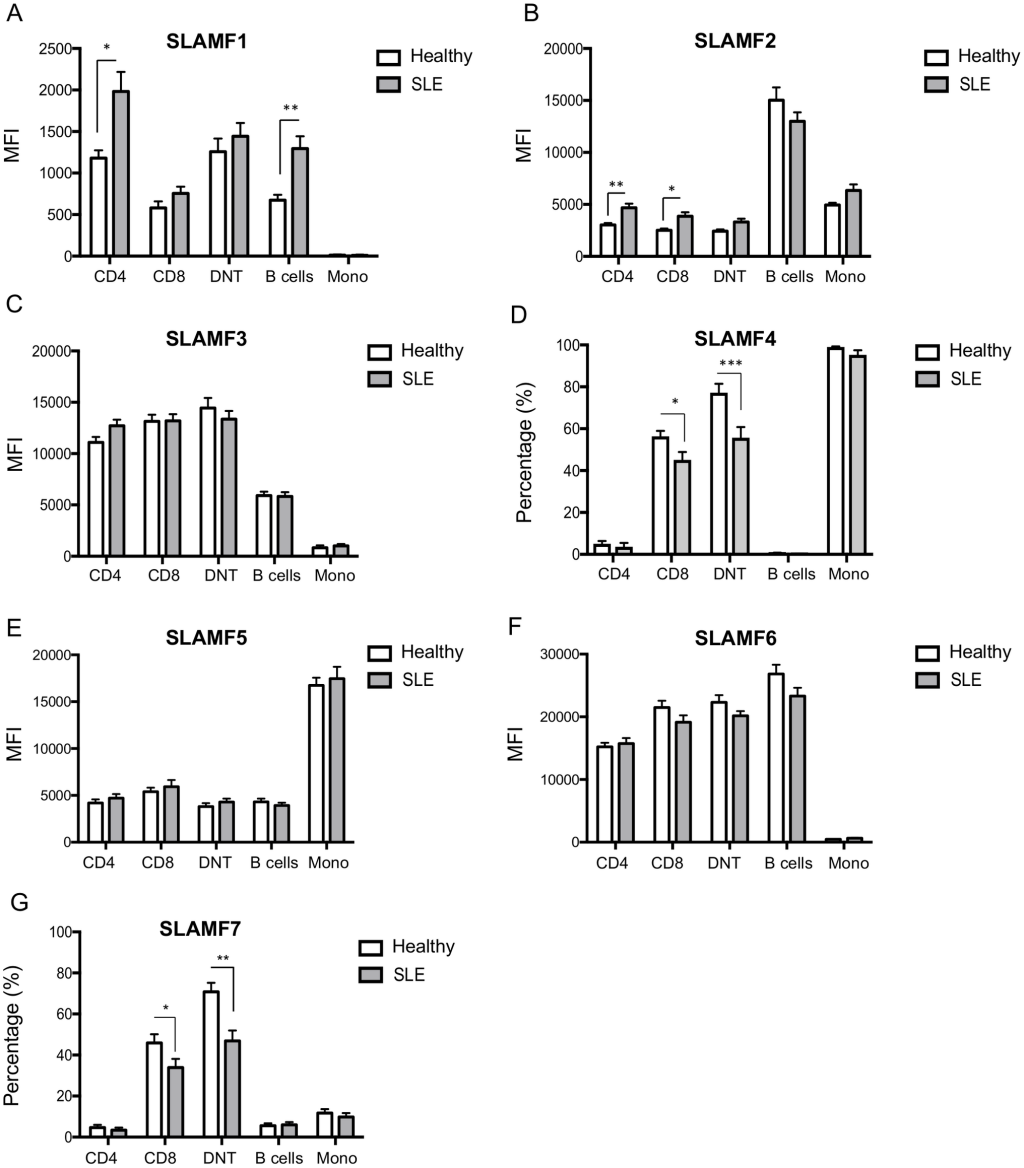
Expression of SLAMF1-7 in lymphocytes and monocytes from healthy individuals and patients with SLE. SLAMF1-7 expression levels were assessed by flow cytometry in PBMC subpopulations from healthy individuals and patients with SLE. Cumulative results of SLAMF expression in CD4+, CD8+, DNT cells, B cells and monocytes are shown for (A) SLAMF1, (B) SLAMF2, (C) SLAMF3, (D) SLAMF4, (E) SLAMF5, (F) SLAMF6 and (G) SLAMF7. Results are expressed as mean MFI ± SEM or mean percentage (%) ± SEM, as indicated.

SLAMF1 expression is more pronounced on T cells. B cells also express SLAMF1, albeit at lower levels compared to T cells, whereas no SLAMF1 expression was seen in monocytes, in agreement with previously reported data (Table 2, Fig 1A and S2A Fig) [3]. Among total T cells, SLAMF1 is primarily expressed by CD4+ and double negative CD3+CD4-CD8-. CD8+ T cells from healthy individuals were also positive for SLAMF1, but levels of expression were lower compared to CD4+ and double negative T cells (Table 2, Fig 1A and S2A Fig).

**Table 2:** Expression of SLAMF1 on peripheral blood T and B lymphocytes, monocytes and on T and B cell differentiated subsets on healthy donors and patients with SLE.

SLAMF1					
	**Healthy Donors (n = 12)**		**SLE patients (n = 15)**		***p value***^b^
	**Mean MFI**^a^	**SEM**	**Mean MFI**	**SEM**	
CD4	1181	92	1982	237	***0*.*040***
CD8	580	79	754	83	*0*.*381*
DNT	1257	159	1443	160	*0*.*667*
B cells	674	65	1296	147	***0*.*008***
Monocytes	12	8	10	8	*0*.*872*
**CD4+ T cells**					
Naïve	175	33	400	102	*0*.*160*
CM	1523	98	2373	198	***0*.*010***
EM	2174	155	3168	243	***0*.*013***
**CD8+ T cells**					
Naïve	24	12	296	67	***0*.*010***
CM	1153	105	1772	106	***0*.*003***
EM	1283	142	1366	150	*0*.*695*
TDEM	205	62	742	131	***0*.*011***
**B cells**	**Healthy Donors (n = 15)**		**SLE patients (n = 12)**		***p value***
	**Mean MFI**	**SEM**	**Mean MFI**	**SEM**	
Transitional	561	74	714	197	*0*.*563*
Naive	515	59	954	157	***0*.*045***
Unswitched Mem	441	49	636	64	*0*.*103*
Switched Mem	267	24	371	50	*0*.*219*
DNB	418	57	651	148	*0*.*330*
Plasmablasts	725	116	570	89	*0*.*549*

^a^ Results are expressed as mean fluorescence intensity (MFI) ± SEM.

^b^ *p* values ≤ 0.05 are considered statistically significant

**Fig 2:**
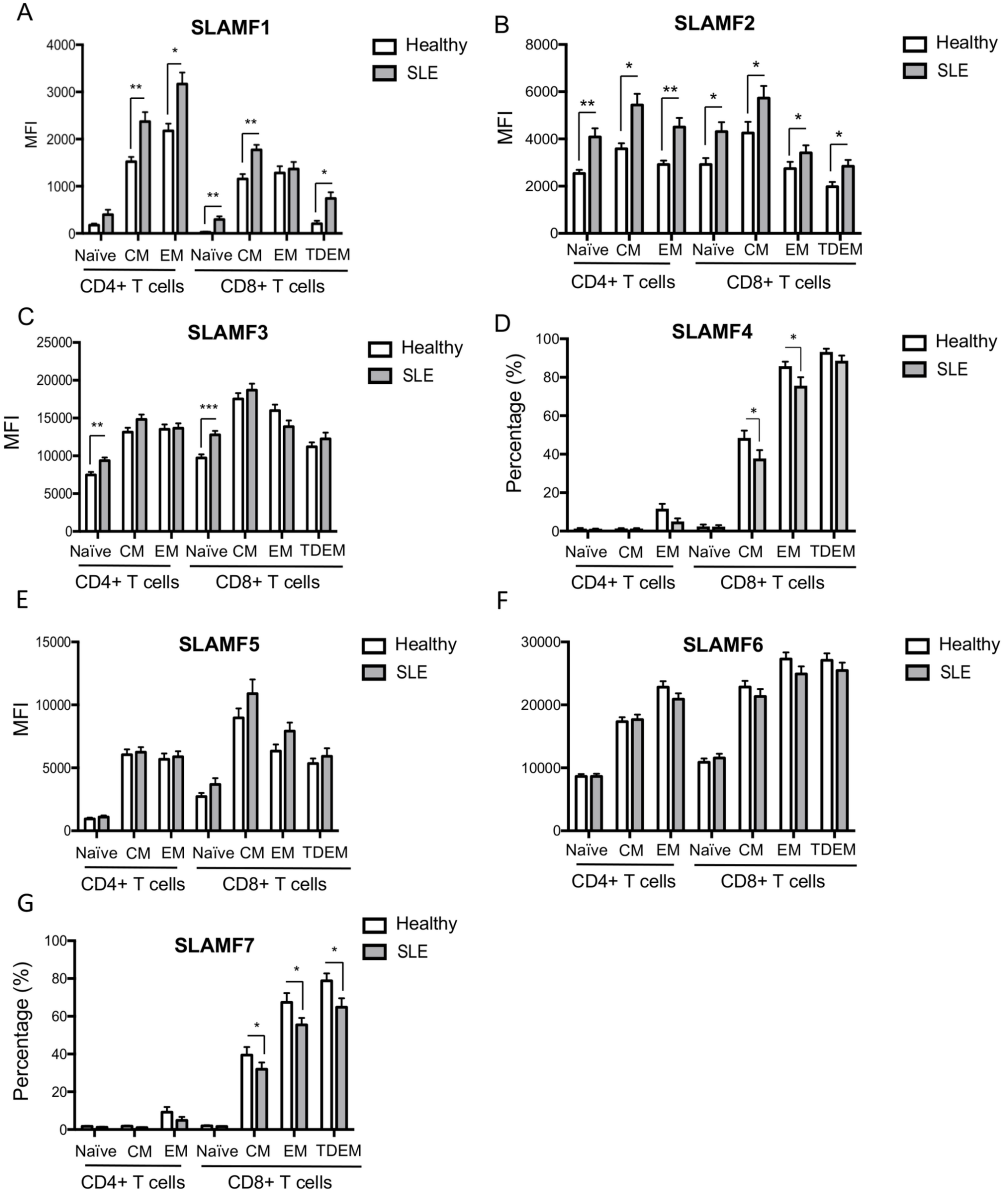
SLAMF1-7 expression in peripheral blood T cell differentiated subpopulations from healthy donors and patients with SLE. Cumulative results of SLAMF expression on CD4+ (naïve, EM and CM) and CD8+ (naïve, EM, CM and TDEM) as assessed by flow cytometry are shown for (A) SLAMF1, (B) SLAMF2, (C) SLAMF3, (D) SLAMF4, (E) SLAMF5, (F) SLAMF6 and (G) SLAMF7. Results are expressed as mean MFI± SEM or mean percentage (%) ± SEM, as indicated.

In patients with SLE, we detected a significant up-regulation of SLAMF1 on the cell-surface of both total T cells and B cells (Table 2, Fig 1A and S2A Fig). Among T cells, SLAMF1 up-regulation was mainly observed on SLE CD4+ T cells, but not on SLE CD8+ or double negative T cells compared to normal donors (Table 2, Fig 1A and S2A Fig). On both SLE and healthy CD4+ T cells, highest SLAMF1 expression was observed on the EM differentiated subset, with SLE CD4+ EM and CM T cells expressing significantly higher SLAMF1 levels compared to healthy controls (Table 2, Fig 2A and S2B Fig).

**Fig 3:**
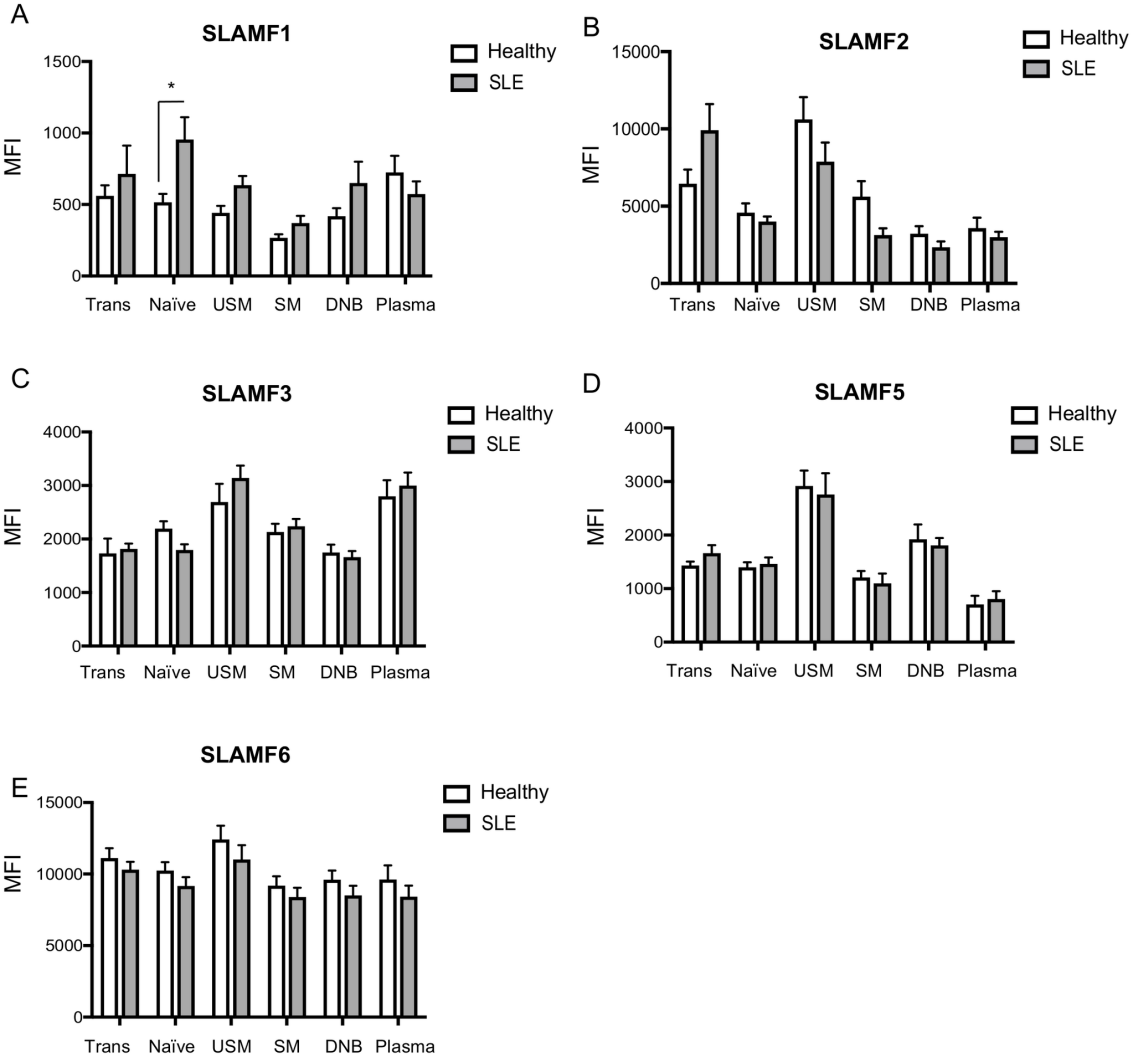
SLAMF1-7 expression in peripheral blood B cell differentiated subpopulations from healthy donors and patients with SLE. Cumulative results of SLAMF expression on transitional, naïve, unswitched memory, switched memory, double negative B cells and plasmablasts are shown for (A) SLAMF1, (B) SLAMF2, (C) SLAMF3, (D) SLAMF5 and (E) SLAMF6. Results are expressed as mean MFI ± SEM

Increased SLAMF1 expression was also seen on SLE naïve, EM and TDEM CD8+ T cells compared to healthy donors (Table 2, Fig 2A and S2B Fig). We then assessed the expression profile of SLAMF1 on the cell surface of peripheral blood B cells subsets in SLE patients and controls. As previously reported, SLAMF1 expression tended to diminish as B cells progress into unswitched and switched memory differentiated subset, with switched memory B cells presenting the lowest levels of expression of SLAMF1[3]. A significant increase in SLAMF1 expression was evident in total SLE B cells and was more pronounced in the naïve B cell compartment (Table 2, Fig 3A and S2C Fig).

We did not establish any correlation between SLAMF1 expression levels and disease activity or specific disease manifestations in our cohort of SLE patients, although a higher number of study subjects may be needed in order to be able to draw any definitive conclusions with respect to clinical implications.

### SLAMF2

Expression of SLAMF2 characterizes all peripheral blood hematopoietic cells. Highest SLAMF2 expression (two to three-fold difference compared to T cells and monocytes) was observed on B cells on both healthy controls and patients with SLE. A tendency towards decreased SLAMF2 expression was documented on SLE total B cells, but the difference did not reach statistical significance (Table 3, Fig 1B and S3A Fig).

**Table 3:** Expression of SLAMF2 on peripheral blood T and B lymphocytes, monocytes and on T and B cell differentiated subsets on healthy donors and patients with SLE.

SLAMF2					
	**Healthy Donors (n = 12)**		**SLE patients (n = 15)**		***p value***^b^
	**Mean MFI**^a^	**SEM**	**Mean MFI**	**SEM**	
CD4	2899	194	4929	497	***0*.*009***
CD8	2328	159	4023	458	***0*.*015***
DNT	2308	169	3606	388	*0*.*028*
B cells	15022	1235	12997	864	*0*.*182*
Monocytes	4945	206	6338	581	*0*.*098*
**CD4+ T cells**					
Naïve	2497	144	4346	457	***0*.*010***
CM	3319	212	5689	600	***0*.*010***
EM	2646	175	4693	517	***0*.*010***
**CD8+ T cells**					
Naïve	2427	148	4662	527	***0*.*008***
CM	3361	207	5803	614	***0*.*010***
EM	2299	154	3414	371	***0*.*020***
TDEM	1574	111	2983	351	***0*.*010***
**B cells**	**Healthy Donors (n = 13)**		**SLE patients (n = 7–9)**		***p value***
	**Mean MFI**	**SEM**	**Mean MFI**	**SEM**	
Transitional	6455	919	9921	1684	*0*.*320*
Naive	4579	608	4003	326	*0*.*720*
Unswitched Mem	10618	1435	7876	1239	*0*.*584*
Switched Mem	5620	998	3126	445	*0*.*320*
DNB	3223	480	2350	364	*0*.*584*
Plasmablasts	3588	677	2998	343	*0*.*720*

^a^ Results are expressed as mean fluorescence intensity (MFI) ± SEM.

^b^ *p* values ≤ 0.05 are considered statistically significant

On the contrary, SLAMF2 was significantly up-regulated on the cell-surface of both SLE CD4+ and CD8+ T cells (Table 3, Fig 1B and S3A Fig). A more detailed analysis of SLAMF2 levels on CD4+ and CD8+ T cells subsets revealed that SLAMF2 expression is independent of the T cells differentiation status. Indeed, SLAMF2 expression levels are comparable among naïve, CM, EM and TDEM CD4+ or CD8+ T cells. Interestingly, differences in expression level of SLAMF2 between SLE and healthy controls are observed in most CD4+ and CD8+ T cells differentiated subsets (Table 3, Fig 2B and S3B Fig). No differences were observed among SLE patients and controls when we assessed the expression of SLAMF2 on the cell surface of differentiated peripheral blood B cell subsets (Table 3, Fig 3B and S3C Fig).

### SLAMF3

SLAMF3 is expressed on almost all hematopoietic cells, with the exception of CD14+ monocytes. SLAMF3 is highly expressed on T cells from both healthy donors and SLE patients. B cells are also positive for SLAMF3, albeit levels of expression are lower compared to that of T cells (Fig 1C and S4A Fig).

In a very small cohort of SLE patients, a slightly increased expression of SLAMF3 has been described on SLE CD4+ T cells compared to healthy controls [20]. In our cohort of patients, we observed only a slight non-statistically significant upregulation of SLAMF3 on the cell surface of CD4+ T cells from patients with SLE compared to normal individuals whereas no differences were documented for CD8+, double negative T cells and B cells among SLE patients and controls (Fig 1C and S4A Fig). A more careful analysis of SLAMF3 expression in CD4+ and CD8+ T cells differentiated subsets revealed a SLAMF3 up-regulation on the cell surface of naïve SLE CD4+ and CD8+ T cells (Fig 2C and S4B Fig). In both SLE patients and healthy donors, SLAMF3 is up-regulated as CD4+ and CD8+ T cells progress from naïve to CM and EM differentiated status. However, no differences were noted regarding SLAMF3 expression levels in CM and EM CD4+ and CD8+ T cells among patients with SLE and healthy controls (Fig 2C and S4B Fig).

Further analysis of SLAMF3 levels on naïve, unswitched memory, switched memory and double negative B cells also revealed no differences between normal controls and SLE patients (Fig 3C and S4C Fig).

### SLAMF4

Presence of SLAMF4 mainly characterizes CD8+ T cells, DNT cells and monocytes. A small percentage (approximately 5%) of CD4+ T cells is also positive for SLAMF4, whereas B cells are SLAMF4 negative (Table 4, Fig 1D and S5A Fig).

**Table 4:** Expression of SLAMF4 on peripheral blood T and B lymphocytes, monocytes and on T cell differentiated subsets on healthy donors and patients with SLE.

SLAMF4					
	Healthy Donors (n = 12–22)		SLE patients (n = 14–25)		*p value*^b^
	Mean (%)^a^	SEM	Mean (%)	SEM	
CD4	5.0	1.4	3.6	1.9	*0*.*925*
CD8	56.4	2.6	45.1	3.7	***0*.*025***
DNT	77.2	4.3	55.7	5.0	***0*.*0000020***
B cells	0.6	0.1	0.2	0.1	*0*.*944*
Monocytes	99.0	0.3	95.3	2.2	*0*.*856*
**CD4+ T cells**					
Naïve	1.1	0.4	0.9	0.3	*1*.*000*
CM	1.3	0.3	1.2	0.4	*1*.*000*
EM	11.7	2.4	5.0	1.6	*0*.*232*
**CD8+ T cells**					
Naïve	2.5	0.9	2.3	0.8	*1*.*000*
CM	48.4	3.9	37.8	4.4	***0*.*014***
EM	85.8	2.4	75.7	4.4	***0*.*020***
TDEM	93.2	1.7	88.6	2.6	*0*.*563*

^a^ Results are expressed as mean percentage (%) ± SEM.

^b^ *p* values ≤ 0.05 are considered statistically significant

SLAMF4 levels increased over CD8+ T cells differentiation in healthy donors: approximately 48% of CM CD8+ T cells and almost all EM and TDEM CD8+ T cells are positive for SLAMF4, whereas naïve CD8+ T cells are SLAMF4 negative cells (Table 4, Fig 2D and S5B Fig). Among CD4+ T cells, naïve and CM are SLAMF4 negative, while approximately 10% of the EM differentiated CD4+ T cells express SLAMF4 (Table 4 Fig 2D and S5B Fig). As previously described [22], the frequency of CD8+SLAMF4+ T cells is significantly reduced in patients with SLE. This reduction is mostly apparent on CD8+ CM and EM SLE T cells (Table 4, Fig 2D and S5B Fig). In addition, the frequency of SLAMF4+CD3+CD4-CD8- double negative cells is decreased in the peripheral blood of patients with SLE (Table 4, Fig 1D and S5A Fig).

### SLAMF5

All circulating hematopoietic cells express SLAMF5, with highest levels of expression being documented on monocytes (Fig 1E and S6A Fig).

Naïve CD4+ T cells express relatively low levels of SLAMF5, but SLAMF5 increased on the cell surface of CM and EM CD4+ T cells in both healthy donors and patients with SLE (Fig 2E and S6B Fig). Naïve CD8+ T cells display slightly higher levels of SLAMF5 compared to naïve CD4+ T cells and, as in CD4+ T cells, SLAMF5 expression increased over cell differentiation (Fig 2E and S6B Fig). Circulating B cells also express high levels of SLAMF5. Highest levels of SLAMF5 expression among B cells differentiated subsets were seen on unswitched memory B cells (Fig 3D and S6C Fig). We detected no differences on SLAMF5 among patients with SLE and healthy controls on none of the cell populations we examined (Figs 1E, 2E and 3D and S6 Fig).

### SLAMF6

SLAMF6 is strongly expressed on the cell surface of both T cells and B cells, but cannot be found on monocytes (Fig 1F and S7A Fig).

On both CD4+ and CD8+ T cells levels of SLAMF6 progressively increase as cells progress into their differentiation status. Highest levels of SLAMF6 expression are seen among CM and EM subsets of CD4+ and CD8+ T cells (Fig 2F and S7B Fig). Despite previous reports showing that SLAMF6 is up-regulated on the cell surface of SLE CD4+ T cells [20] in a small cohort of SLE patients, we were not able to detect any differences in our cohort among SLE patients and healthy controls. Highest level of SLAMF6 expression was observed on total B cells and its levels appear to remain stable on the differentiated subsets of circulating B cells (Figs 1F and 3E and S7C Fig), without any differences among SLE patients and normal controls.

### SLAMF7

As with SLAMF4, expression of SLAMF7 mainly characterizes CD8+ and DNT cells, and is present only on a very small proportion (less than 5%) of CD4+ T cells (Table 5, Fig 1G and S8A Fig).

**Table 5:** Expression of SLAMF7 on peripheral blood T and B lymphocytes, monocytes and on T cell differentiated subsets on healthy donors and patients with SLE.

SLAMF7					
	Healthy Donors (n = 11–21)		SLE patients (n = 15–26)		*p value*^b^
	Mean (%)^a^	SEM	Mean (%)	SEM	
CD4	4.7	1.3	2.9	1.1	*0*.*663*
CD8	54.7	3.3	38.8	3.9	***0*.*019***
DNT	70.8	4.5	47.8	4.2	***0*.*006***
B cells	5.5	1.2	5.7	1.1	*0*.*959*
Monocytes	11.7	1.9	10.9	2.3	*0*.*959*
**CD4+ T cells**					
Naïve	1.7	0.2	1.2	0.2	*0*.*257*
CM	1.8	0.3	1.1	0.2	*0*.*145*
EM	11.3	3.8	4.2	1.5	*0*.*188*
**CD8+ T cells**					
Naïve	3.1	0.8	3.3	0.8	*0*.*885*
CM	47.5	3.3	33.7	3.0	***0*.*024***
EM	76.6	2.9	63.4	3.3	***0*.*029***
TDEM	86.9	2.9	73.4	3.6	***0*.*033***

^a^ Results are expressed as mean percentage (%) ± SEM.

^b^ *p* values ≤ 0.05 are considered statistically significant

Its levels of expression are low on naïve CD8+ T cells. Approximately 3% of naïve CD8+ T cells express SLAMF7 (Table 5, Fig 2G and S8B Fig). As naïve CD8+ T cells differentiate into CM and EM cells, SLAMF7 expression progressively increases. In healthy controls, approximately 40% of CM CD8+ T cells, 70% of EM CD8+ and 80% of TDEM CD8+ T cells are SLAMF7 positive (Table 5, Fig 2G and S8B Fig). With respect to CD4+ T cells, less than 10% of healthy EM CD4+ T cells express SLAMF7. In SLE we observed a significant decrease in the frequency of SLAMF7 expressing double negative and CD8+ T cells populations (Table 5, Fig 1G and S8A Fig). Among CD8+ T cells, decreased SLAMF7 expression was evident on CM cells, and was even more apparent on SLE EM and TDEM CD8+ T cells, compared to normal controls (Table 5, Fig 2G and S8B Fig). As far as B cells are concerned, we were not able to detect SLAMF7 on the cell surface of peripheral blood total B cells isolated from healthy controls or patients with SLE, despite previously published reports (Table 5, Fig 1G and S8A Fig) [23].

## Discussion

Adaptive immunity plays an important role in immunopathogenesis of SLE. In this context, T and B cells play an important role in this process [1, 2]. Full T cell and B cell activation requires recognition of antigen via the cognate antigenic receptor (TCR and BCR respectively), followed by a second signal provided via co-stimulatory receptors. The best characterized co-stimulatory pathways for T cell activation involves the engagement of CD28 to CD80 and CD86, and for B cell activation the interaction of CD40L to CD40. However, other molecules may also contribute to optimal T cell and/or B cell activation and differentiation. During recent years, the SLAMF receptors have emerged as important immunoregulatory molecules implicated in autoimmunity. Their involvement in SLE was initially suggested by genome-wide linkage analysis studies performed in SLE affected families in which the 1q23 locus on chromosome 1, where the *SLAMF* gene cluster is located, was identified as a lupus susceptibility locus. Moreover, data from recent studies have unveiled the existence of SNPs and polymorphisms of SLAMF molecules associated with SLE and/or specific lupus manifestations [9, 10, 24]. Aberrant expression of various SLAMF molecules has also been reported in SLE patients [18, 22, 25, 26].

In this study we performed a systematic analysis of the expression pattern of SLAMF1-7 on peripheral blood cells involved in adaptive immunity. Therefore, we examined the expression of SLAMF1-7 on monocytes, T cells, B cells and their respective differentiated subsets isolated from the peripheral blood of patients with SLE. Our findings are of particular interest regarding the expression levels of SLAMF1, SLAMF2, SLAMF4 and SLAMF7. We found a significant up-regulation of SLAMF1 on the cell surface of both T cells and B cells in patients with SLE, in agreement with previously published data [15]. In SLE T cells, up-regulation in SLAMF1 levels was mostly evident in differentiated CD4+ CM and EM subsets, yet a tendency towards increased SLAMF1 levels was already apparent in the naïve CD4+ compartment. SLE B cells presented with higher SLAMF1 levels compared to healthy controls, a difference that was mostly evident in the naïve B cell compartment. Although the pathophysiological significance of this finding in SLE remains to be determined, SLAMF1 engagement on healthy human B cells has been shown to promote B cell proliferation and Ig production [27]. Regarding T cells, SLAMF1 ligation on human T cell clones has been shown to induce IL-2-independent, cyclosporine A-sensitive proliferation and to promote IFN-gamma production [28].

SLAMF2 is structurally different compared to other SLAMF members in the sense that it is a glycophosphatidylinositol (GPI) membrane anchor without cytoplasmic tail. Despite the lack of an intracellular domain, SLAMF2 is capable of eliciting downstream signaling upon interaction with SLAMF4 or CD2 [29], although how signaling is mediated remains unclear. An increase in SLAMF2 mRNA levels has been previously described for SLE CD4+ T cells [30]. We found a significant up-regulation of SLAMF2 on the cell surface of SLE CD4+ and CD8+ T cells that was evident on all differentiated subsets, even naïve CD4+ and CD8+ T cells. It has been proposed that the primary function of the SLAMF2:CD2 interaction is to facilitate adhesion and to secure the distance between T cells and antigen presenting cells for optimal antigen presentation [31]. Moreover, SLAMF2 constitutes a central component of the lipid rafts. SLAMF2 ligation has been shown to be able to amplify early TCR-initiated responses by facilitating actin cytoskeleton reorganization and recruitment of associated lipid rafts to the TCR associated activation cap [32]. Freshly isolated SLE T cells are characterized by faster actin polymerization kinetics compared to normal T cells, as well as by the presence of pre-clustered lipid rafts that are enriched for activated Syk and the common γ chain of the Fcε receptor (FcεRγ) [33]. Alterations described in the lipid raft signaling machinery in SLE T cells may contribute to the hyperactivity that characterizes lupus T cells [33]. Whether increased SLAMF2 expression facilitates lipid raft clustering on SLE T cells or whether targeting SLAMF2 can disrupt lipid raft formation, therefore correcting aberrant signaling responses in lupus, remains to be examined.

In patients with SLE, the expression profile pattern of SLAMF4 and SLAMF7 is of particular interest in the CD8+ T cell compartment. Normally, both molecules increase on the cell surface of healthy CD8+ T cells as they progress into differentiation to CM and EM cells. However, the frequency of SLAMF4+ and SLAMF7+ CD8+ T cells is significantly reduced in patients with SLE. Both SLAMF4 and SLAMF7 characterize CD8+ T cells with cytotoxic capacity [22, 34]. Additionally, in patients with SLE the viral-specific antigenic responses of SLAMF4+ and SLAMF7+ CD8+ T cells are defective [22, 35, 36]. The loss of the SLAMF4+ and SLAMF7+ memory CD8+ T cell population may account for the increased rate of infections, the leading cause of mortality in SLE.

For SLAMF3, SLAMF5 and SLAMF6 we were not able to identify any substantial differences in expression levels among healthy controls and patients with SLE. However, as was illustrated for SLAMF3 [16], the possibility remains that their function is not intact in lupus. The intracellular region of SLAMF molecules, with the exception of SLAMF2, contains at least one ITSM sequence that binds with high affinity to either SAP or EAT-2. Reduced, but not absent, SAP levels have been described for SLE T cells and this decrease may interfere with proper signal transduction upon SLAMF:SLAMF engagement [37].

SLAMF molecules could present potential therapeutic targets in autoimmunity. SLAMF3 co-engagement on CD4+ T cells with the use of a specific monoclonal antibody, enhances CD4+ T cells sensitivity to IL-2 and favors regulatory T-cell polarization [16]. Moreover, despite reduced SLAMF7 levels in lupus CD8+ T cells, targeting SLAMF7 allowed to enhance anti-viral cytotoxic responses in patients with SLE [34]. As the need for new drugs and treatment strategies in SLE remains mandatory, further understanding the potential involvement of SLAMF molecules in lupus immunopathogenesis may lead to the development of novel therapeutic options.

## Supporting information

S1 Fig Gating strategy. Representative flow panels and gating strategy of peripheral blood mononuclear cells isolated from healthy controls and patients with SLE. (A) T cells: CD3+CD19-; B cells: CD3-CD19+; CD4+ T cells: CD3+CD4+CD19-; CD8+ T cells: CD3+CD8+CD19-; double negative T cells (DNT): CD3+CD4-CD8-; monocytes: CD14+; naïve CD4+ or CD8+ T cells: CCR7+CD45RA+; central Memory (CM) CD4+ or CD8+ T cells: CCR7+CD45RA-; effector memory (EM) CD4+ or CD8+ T cells: CCR7-CD45RA-; terminally differentiated effector memory CD8+ T cells (TDEM): CCR7-CD45RA+. (B) Lymphocytes are defined as in (A) (upper panel). B cells are defined as CD19+CD20+ cells. Naïve B cells: IgD+CD27-; unswitched memory B cells (USM): IgD+CD27+; switched memory B cells (SM): IgD-CD27+; double negative B cells (DNB): IgD-CD27-; transitional B cells: IgD+CD27-CD24^hi^CD38^hi^; plasmablasts: IgD-CD27+CD24-CD38+.
(TIF)

S2 Fig Expression of SLAMF1 on peripheral blood T and B lymphocytes, monocytes and on T and B cell differentiated subsets on healthy donors and patients with SLE. SLAMF1 expression was assessed by flow cytometry on (A) CD4+, CD8+, Double negative T cells (DNT), B cells and monocytes, (B) T cell differentiated subsets and (C) B cell differentiated subsets. CM = central memory; EM = effector memory; TDEM = Terminally Differentiated Effector Memory; USM = unswitched memory; DNB = double negative B cells.
(TIF)

S3 Fig Expression of SLAMF2 on peripheral blood T and B lymphocytes, monocytes and on T and B cell differentiated subsets on healthy donors and patients with SLE. SLAMF2 expression was assessed by flow cytometry on (A) CD4+, CD8+, Double negative T cells (DNT), B cells and monocytes, (B) T cell differentiated subsets and (C) B cell differentiated subsets. CM = central memory; EM = effector memory; TDEM = Terminally Differentiated Effector Memory; USM = unswitched memory; DNB = double negative B cells.
(TIF)

S4 Fig Expression of SLAMF3 on peripheral blood T and B lymphocytes, monocytes and on T and B cell differentiated subsets on healthy donors and patients with SLE. SLAMF3 expression was assessed by flow cytometry on (A) CD4+, CD8+, Double negative T cells (DNT), B cells and monocytes, (B) T cell differentiated subsets and (C) B cell differentiated subsets. CM = central memory; EM = effector memory; TDEM = Terminally Differentiated Effector Memory; USM = unswitched memory; DNB = double negative B cells.
(TIF)

S5 Fig Expression of SLAMF4 on peripheral blood T and B lymphocytes, monocytes and on T cell differentiated subsets on healthy donors and patients with SLE. SLAMF4 expression was assessed by flow cytometry on (A) CD4+, CD8+, Double negative T cells (DNT), B cells and monocytes, (B) T cell differentiated subsets. CM = central memory; EM = effector memory; TDEM = Terminally Differentiated Effector Memory; USM = unswitched memory; DNB = double negative B cells.
(TIF)

S6 Fig Expression of SLAMF5 on peripheral blood T and B lymphocytes, monocytes and on T and B cell differentiated subsets on healthy donors and patients with SLE. SLAMF5 expression was assessed by flow cytometry on (A) CD4+, CD8+, Double negative T cells (DNT), B cells and monocytes, (B) T cell differentiated subsets and (C) B cell differentiated subsets. CM = central memory; EM = effector memory; TDEM = Terminally Differentiated Effector Memory; USM = unswitched memory; DNB = double negative B cells.
(TIF)

S7 Fig Expression of SLAMF6 on peripheral blood T and B lymphocytes, monocytes and on T and B cell differentiated subsets on healthy donors and patients with SLE. SLAMF6 expression was assessed by flow cytometry on (A) CD4+, CD8+, Double negative T cells (DNT), B cells and monocytes, (B) T cell differentiated subsets and (C) B cell differentiated subsets. CM = central memory; EM = effector memory; TDEM = Terminally Differentiated Effector Memory; USM = unswitched memory; DNB = double negative B cells.
(TIF)

S8 Fig Expression of SLAMF7 on peripheral blood T and B lymphocytes, monocytes and on T cell differentiated subsets on healthy donors and patients with SLE. SLAMF7 expression was assessed by flow cytometry on (A) CD4+, CD8+, Double negative T cells (DNT), B cells and monocytes, (B) T cell differentiated subsets. CM = central memory; EM = effector memory; TDEM = Terminally Differentiated Effector Memory; USM = unswitched memory; DNB = double negative B cells.
(TIF)
