# Supplementary figures and images for: Expression patterns of signaling lymphocytic activation molecule family members in peripheral blood mononuclear cell subsets in patients with systemic lupus erythematosus
Source: PLoS One. 2017 Oct 11;12(10):e0186073. doi: 10.1371/journal.pone.0186073 (PMC5636110; doi:10.1371/journal.pone.0186073)

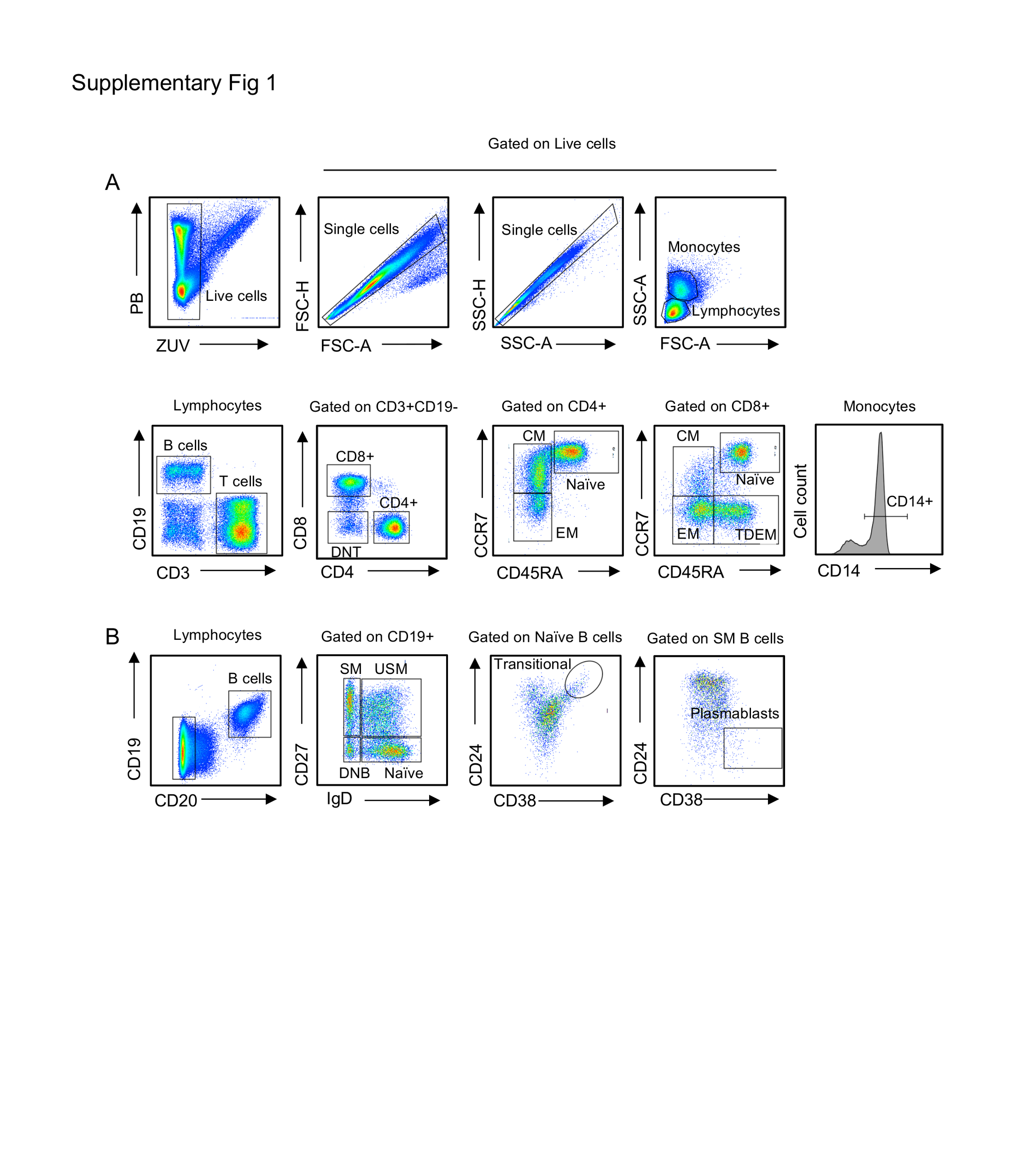

Supplement: S1 Fig — Representative flow panels and gating strategy of peripheral blood mononuclear cells isolated from healthy controls and patients with SLE. (A) T cells: CD3+CD19-; B cells: CD3-CD19+; CD4+ T cells: CD3+CD4+CD19-; CD8+ T cells: CD3+CD8+CD19-; double negative T cells (DNT): CD3+CD4-CD8-; monocytes: CD14+; naïve CD4+ or CD8+ T cells: CCR7+CD45RA+; central Memory (CM) CD4+ or CD8+ T cells: CCR7+CD45RA-; effector memory (EM) CD4+ or CD8+ T cells: CCR7-CD45RA-; terminally differentiated effector memory CD8+ T cells (TDEM): CCR7-CD45RA+. (B) Lymphocytes are defined as in (A) (upper panel). B cells are defined as CD19+CD20+ cells. Naïve B cells: IgD+CD27-; unswitched memory B cells (USM): IgD+CD27+; switched memory B cells (SM): IgD-CD27+; double negative B cells (DNB): IgD-CD27-; transitional B cells: IgD+CD27-CD24hiCD38hi; plasmablasts: IgD-CD27+CD24-CD38+. (TIF) [file pone.0186073.s001.tif]

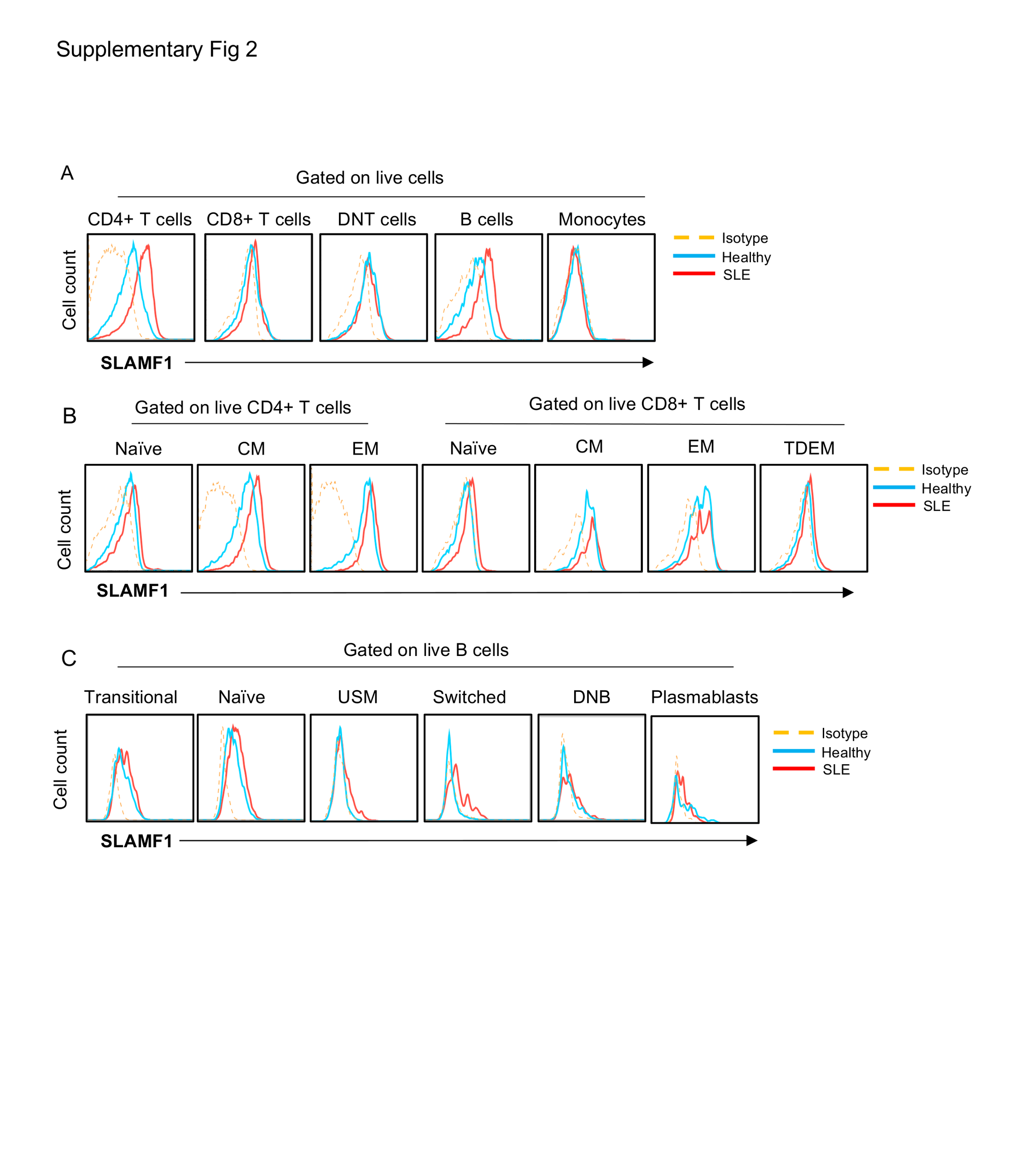

Supplement: S2 Fig — SLAMF1 expression was assessed by flow cytometry on (A) CD4+, CD8+, Double negative T cells (DNT), B cells and monocytes, (B) T cell differentiated subsets and (C) B cell differentiated subsets. CM = central memory; EM = effector memory; TDEM = Terminally Differentiated Effector Memory; USM = unswitched memory; DNB = double negative B cells. (TIF) [file pone.0186073.s002.tif]

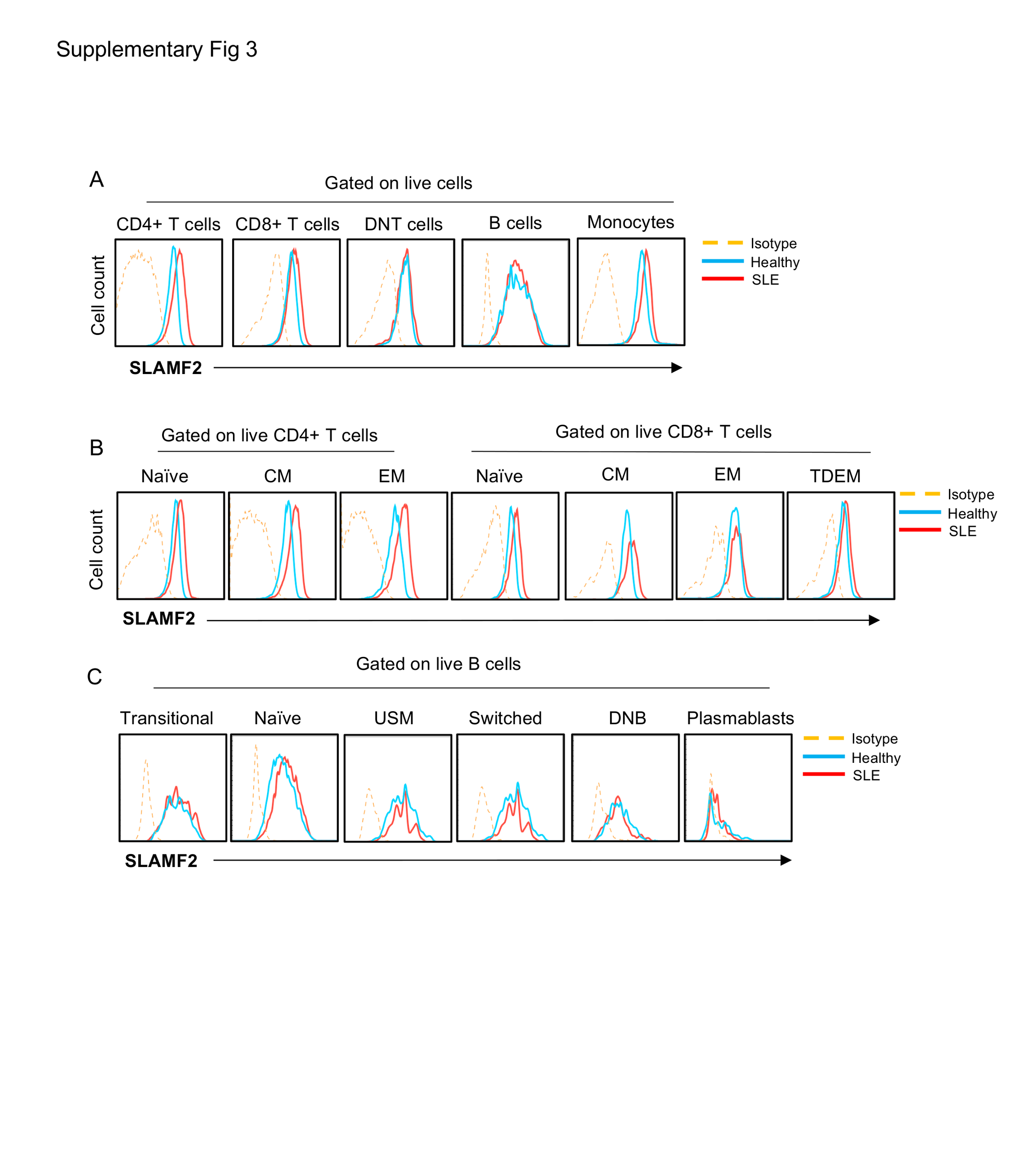

Supplement: S3 Fig — SLAMF2 expression was assessed by flow cytometry on (A) CD4+, CD8+, Double negative T cells (DNT), B cells and monocytes, (B) T cell differentiated subsets and (C) B cell differentiated subsets. CM = central memory; EM = effector memory; TDEM = Terminally Differentiated Effector Memory; USM = unswitched memory; DNB = double negative B cells. (TIF) [file pone.0186073.s003.tif]

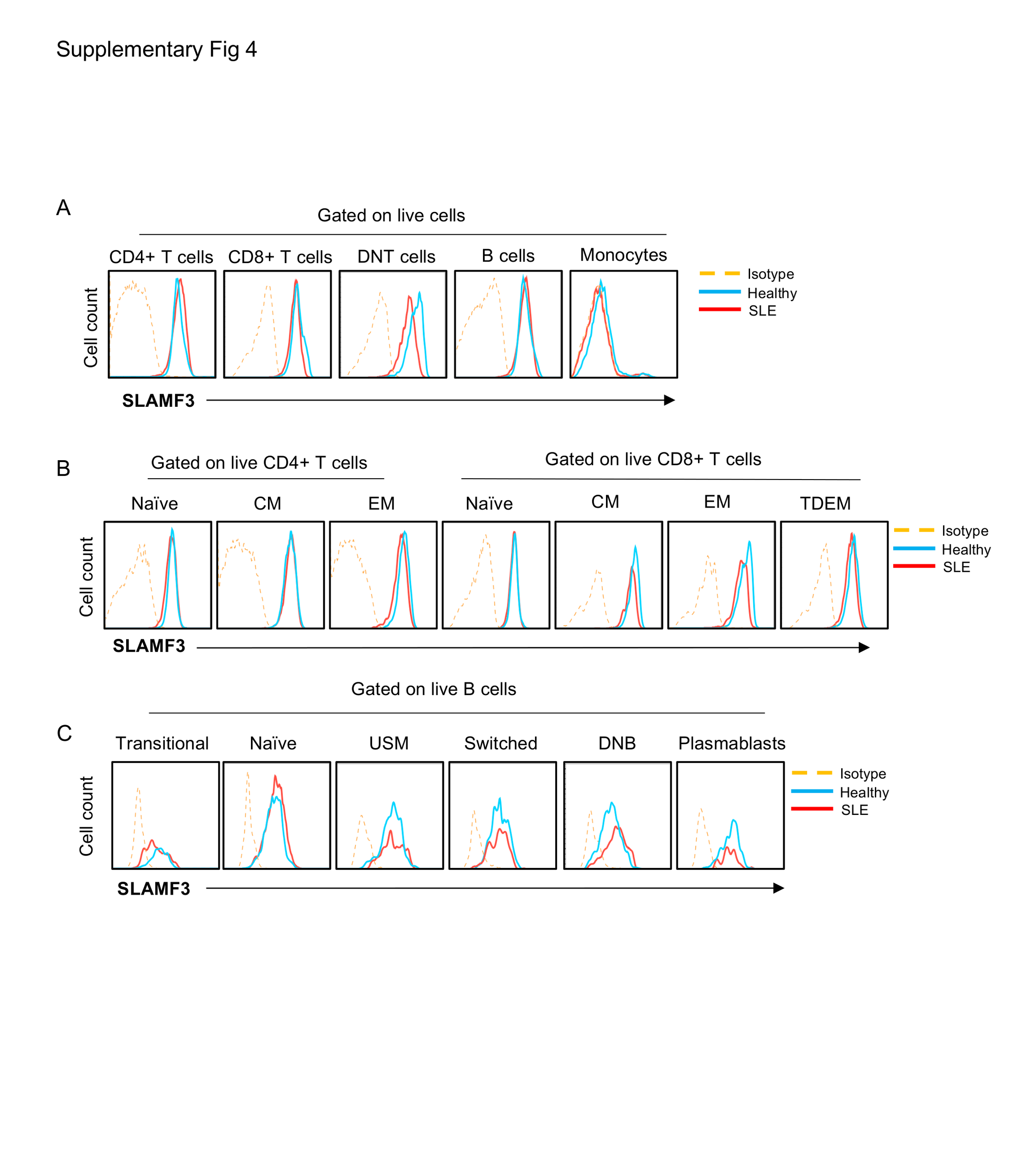

Supplement: S4 Fig — SLAMF3 expression was assessed by flow cytometry on (A) CD4+, CD8+, Double negative T cells (DNT), B cells and monocytes, (B) T cell differentiated subsets and (C) B cell differentiated subsets. CM = central memory; EM = effector memory; TDEM = Terminally Differentiated Effector Memory; USM = unswitched memory; DNB = double negative B cells. (TIF) [file pone.0186073.s004.tif]

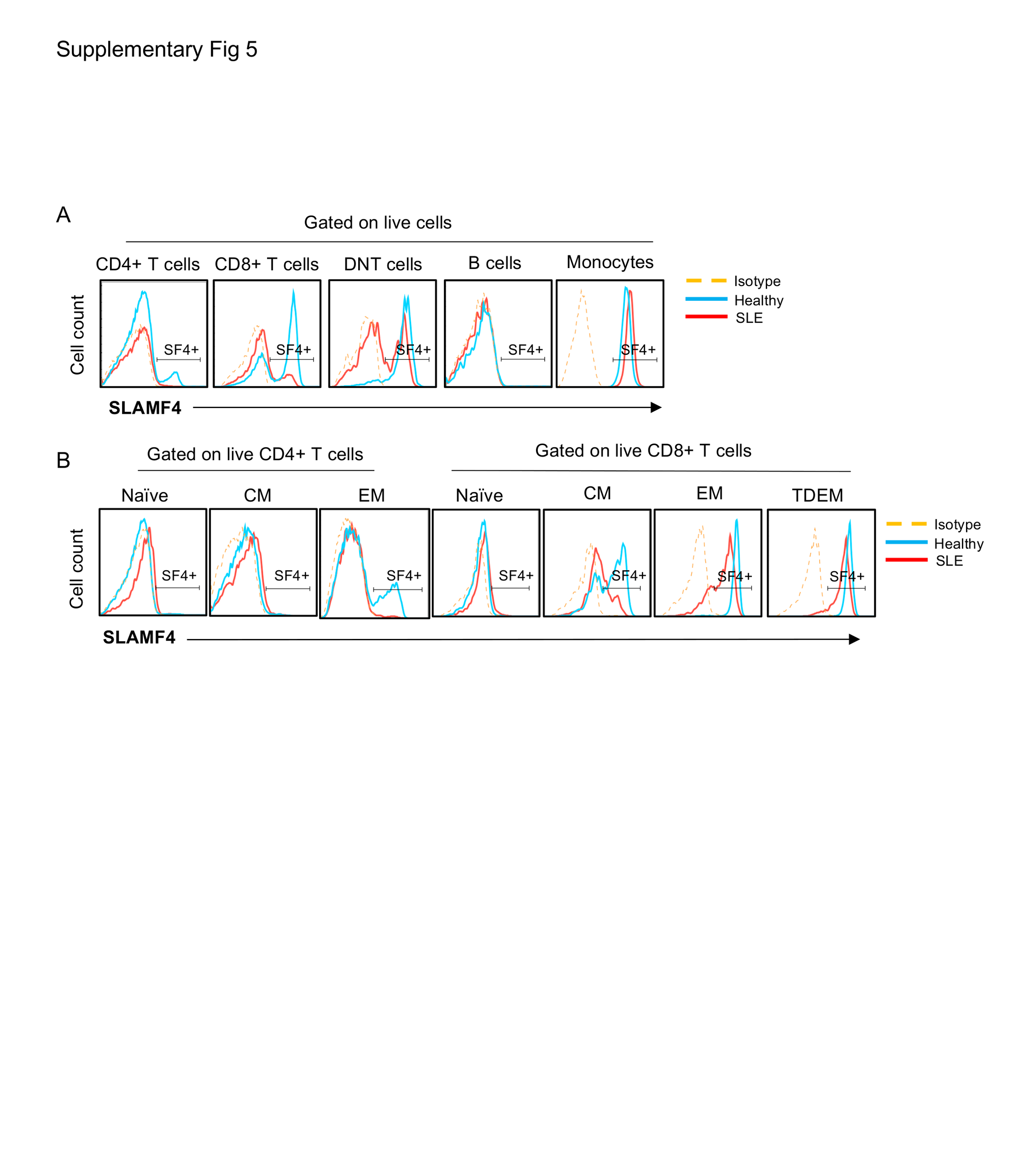

Supplement: S5 Fig — SLAMF4 expression was assessed by flow cytometry on (A) CD4+, CD8+, Double negative T cells (DNT), B cells and monocytes, (B) T cell differentiated subsets. CM = central memory; EM = effector memory; TDEM = Terminally Differentiated Effector Memory; USM = unswitched memory; DNB = double negative B cells. (TIF) [file pone.0186073.s005.tif]

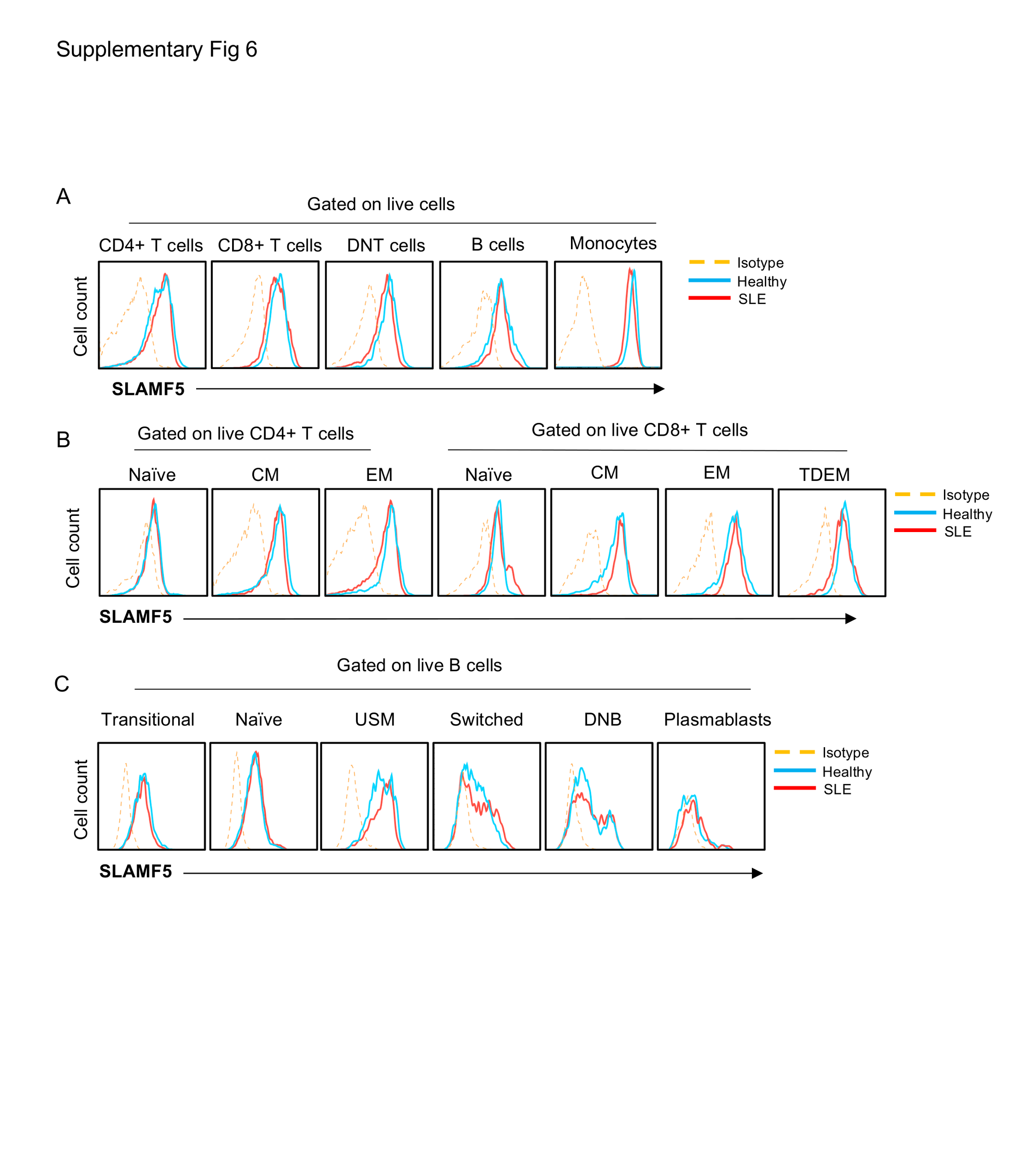

Supplement: S6 Fig — SLAMF5 expression was assessed by flow cytometry on (A) CD4+, CD8+, Double negative T cells (DNT), B cells and monocytes, (B) T cell differentiated subsets and (C) B cell differentiated subsets. CM = central memory; EM = effector memory; TDEM = Terminally Differentiated Effector Memory; USM = unswitched memory; DNB = double negative B cells. (TIF) [file pone.0186073.s006.tif]

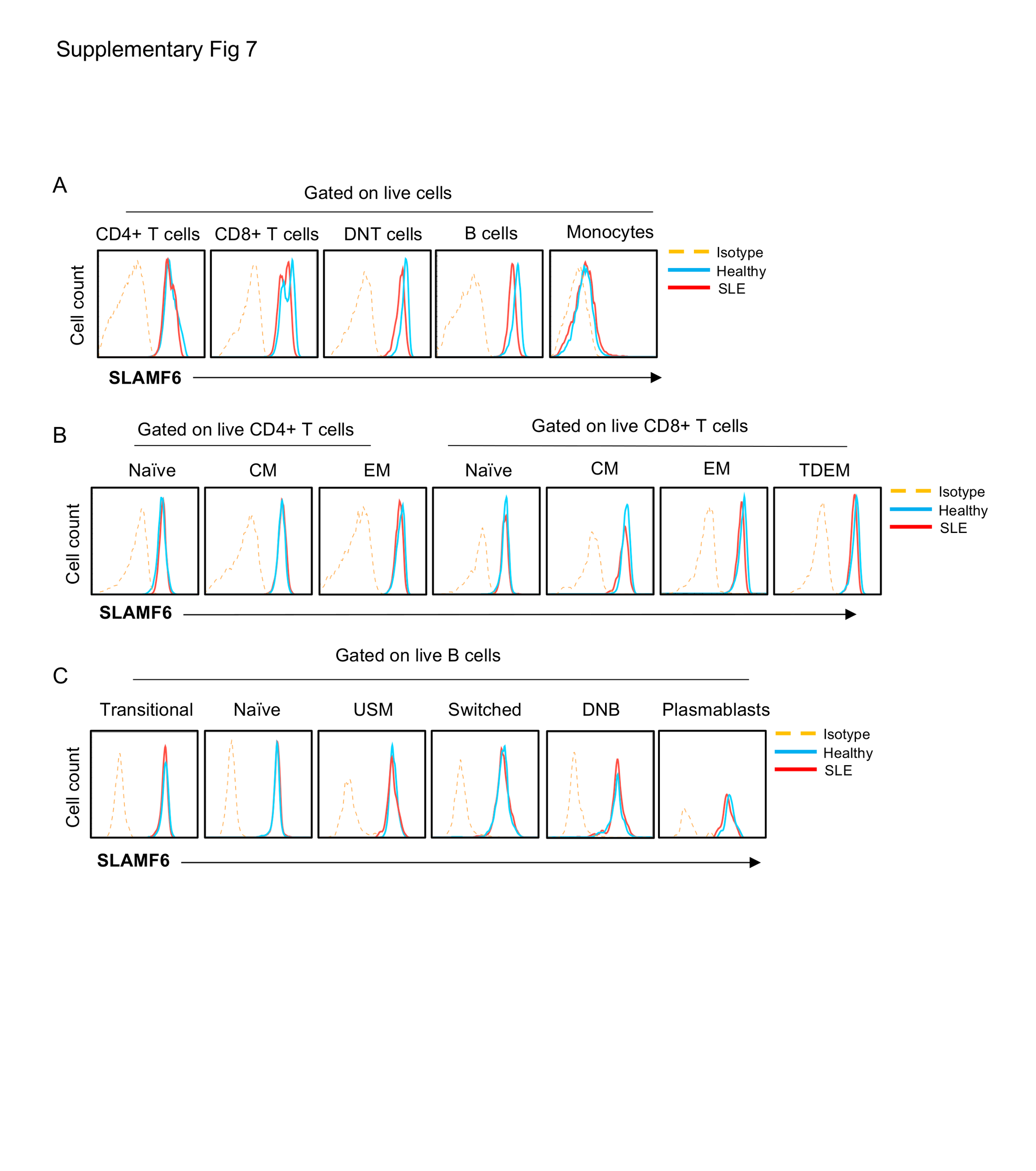

Supplement: S7 Fig — SLAMF6 expression was assessed by flow cytometry on (A) CD4+, CD8+, Double negative T cells (DNT), B cells and monocytes, (B) T cell differentiated subsets and (C) B cell differentiated subsets. CM = central memory; EM = effector memory; TDEM = Terminally Differentiated Effector Memory; USM = unswitched memory; DNB = double negative B cells. (TIF) [file pone.0186073.s007.tif]

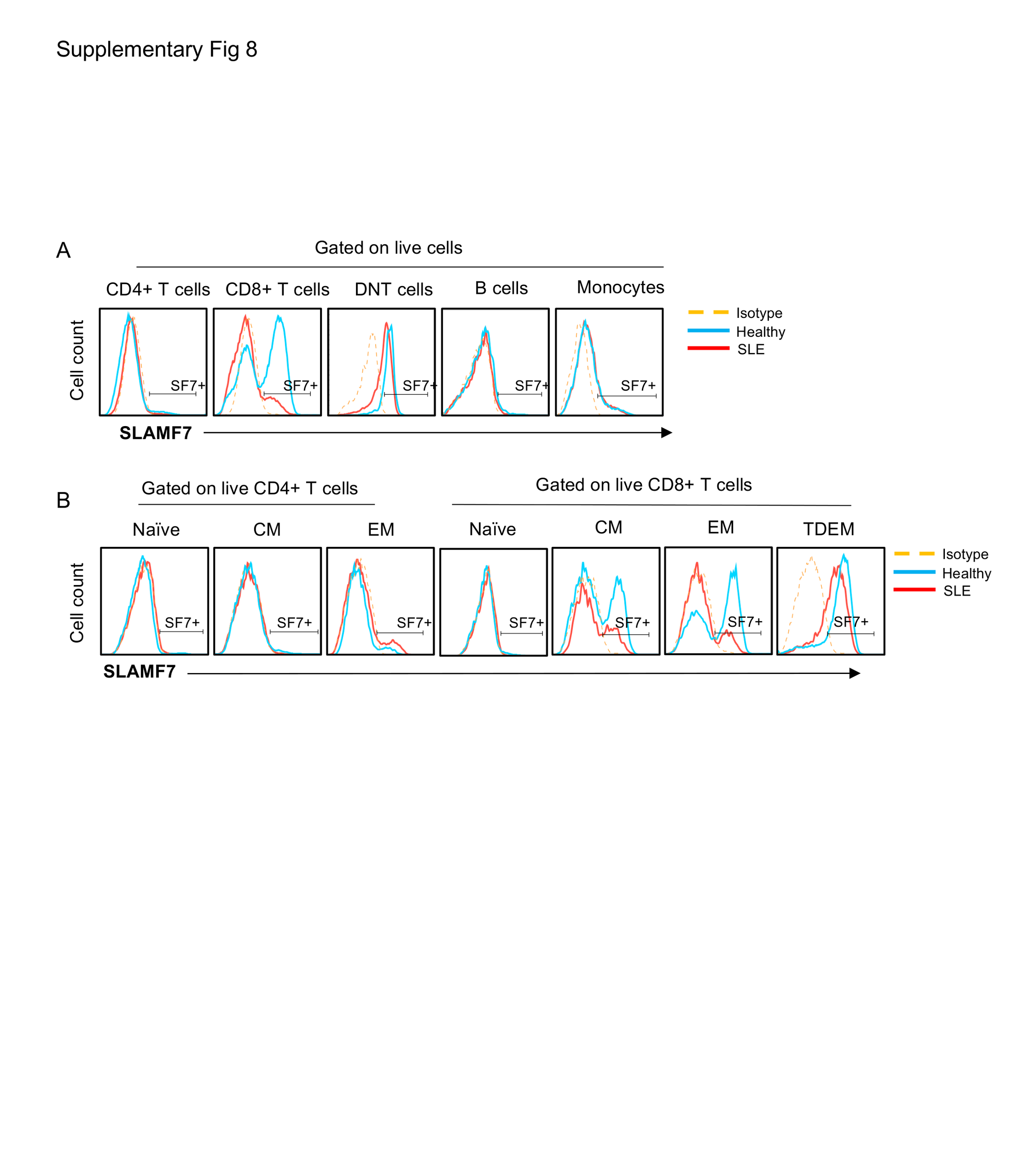

Supplement: S8 Fig — SLAMF7 expression was assessed by flow cytometry on (A) CD4+, CD8+, Double negative T cells (DNT), B cells and monocytes, (B) T cell differentiated subsets. CM = central memory; EM = effector memory; TDEM = Terminally Differentiated Effector Memory; USM = unswitched memory; DNB = double negative B cells. (TIF) [file pone.0186073.s008.tif]
